# AQP5-1364A/C polymorphism and the AQP5 expression influence sepsis survival and immune cell migration: a prospective laboratory and patient study

**DOI:** 10.1186/s12967-016-1079-2

**Published:** 2016-11-21

**Authors:** Katharina Rump, Matthias Unterberg, Lars Bergmann, Agnes Bankfalvi, Anil Menon, Simon Schäfer, André Scherag, Zainab Bazzi, Winfried Siffert, Jürgen Peters, Michael Adamzik

**Affiliations:** 1 Klinik für Anästhesiologie, Intensivmedizin und Schmerztherapie, Universitätsklinikum Knappschaftskrankenhaus Bochum-Langendreer, In der Schornau 55, 45882 Bochum, Germany; 2 Institut für Pharmakogenetik, Universität Duisburg-Essen, Duisburg, Germany; 3 Klinik für Anästhesiologie und Intensivmedizin, Universitätsklinikum and Universität Duisburg-Essen, Essen, Germany; 4 Institut für Pathologie, Universitätsklinikum and Universität Duisburg-Essen, Essen, Germany; 5 Department of Molecular Genetics, Biochemistry and Microbiology, University of Cincinnati, Cincinnati, OH USA; 6 Clinical Epidemiology, Integrated Research and Treatment Center, Center for Sepsis Control and Care (CSCC), Jena University Hospital, Jena, Germany; 7 Klinik für Anästhesiologie, LMU, Munich, Germany

## Abstract

**Background:**

The C-allele of the aquaporin (AQP5) -1364A/C polymorphism is associated with decreased AQP5 expression but increased 30-day survival in patients with severe sepsis. AQP5 expression might affect survival via an impact on cell migration. Consequently, we tested the hypothesis that (1) *Aqp5* knockout (KO) compared to wild type (WT) mice show an increased survival following lipopolysaccharide (LPS) administration, and that (2) AQP5 expression and the AQP5 -1364A/C polymorphism alters immune cell migration.

**Methods:**

We investigated *Aqp5*-KO and wild type mice after intraperitoneal injection of either *E.coli* lipopolysaccharide (LPS, serotype O127:B8, 20 mg/kg) or saline. Furthermore, neutrophils of volunteers with the AA-*AQP5* or AC/CC-*AQP5*- genotype were incubated with 10^−8^ M Chemotactic peptide (fMLP) and their migration was assessed by a filter migration assay. Additionally, AQP5 expression after fMLP incubation was analyzed by RT-PCR and Western blot. Moreover, migration of AQP5 overexpressing Jurkat cells was studied after SDF-1α-stimulation. We used exact Wilcoxon–Mann–Whitney tests; exact Wilcoxon signed-rank tests and the Kaplan–Meier estimator for statistical analysis.

**Results:**

Fifty-six percent of *Aqp5*-KO but only 22% of WT mice survived following LPS-injection. WT mice showed increased neutrophil migration into peritoneum and lung compared to *Aqp5*-KO mice. Target-oriented migration of neutrophils was seen after 0.5 h in AA-genotype cells but only after 1.5 h in AC/CC-genotype cells, with a threefold lower migrating cell count. AQP5 overexpressing Jurkat cells showed a 2.4 times stronger migration compared to native Jurkat cells.

**Conclusion:**

The *AQP5* genotype may influence survival following LPS by altering neutrophil cell migration.

*Trial registration* DRKS00010437. Retrospectively registered 26 April 2016

**Electronic supplementary material:**

The online version of this article (doi:10.1186/s12967-016-1079-2) contains supplementary material, which is available to authorized users.

## Background

Although wide variability exists regarding the outcome from severe sepsis, some of this variability may be caused by genetic variability, as shown in twin and adoption studies [1–4]. A potential candidate gene for investigation is the gene encoding aquaporin (AQP) 5 [5], which mediates key mechanisms of inflammation that prevail in sepsis, including cell migration and proliferation [6], activity of the renin–angiotensin–aldosterone system (RAAS) [7], and the transport of water across biological membranes [8]. Previously, we described a functional, and common (70% AA, 23% AC, 7% CC) single nucleotide (−1364A/C, rs3759129) polymorphism in the *AQP5* gene promoter [7]. Substitution of C for A at position—1364 was associated with a decreased *AQP5* messenger RNA and AQP5 protein expression. It was also a strong, independent prognostic factor for decreased 30-day mortality in patients with severe sepsis [9]. The estimated hazard ratio of nearly 4 for the AC/CC-genotypes compared with the homozygous AA genotype not only suggests that the C-allele of the *AQP5*-1364A/C polymorphism may have important effects on the AQP5 expression in severe sepsis, but also that there is a potential relevance of the AQP5 expression in severe sepsis. However, the underlying molecular and pathophysiological alterations linking a decreased AQP5 expression in C allele carriers to 30-day mortality is unknown.

In the search for these mechanisms, our previous research [7] suggested an impact of a decreased AQP5 expression in C-allele carriers on sepsis survival via its influence on the renin–angiotensin–aldosterone system, which however could not be confirmed [9]. Therefore, our research focused on further possible mechanisms. Since AQP5 expression also mediates cell migration, which is yet another inflammatory key mechanism and involves transient formation of membrane protrusions (lamellipodia and membrane ruffles) at the migrating cell's leading edge [6], we speculated that the AQP5 expression might influence sepsis survival due to its influence on immune cell migration. During cell migration aquaporins facilitate water influx through the cell membrane and cause a local hydrostatic pressure causing cell membrane expansion [6]. This expansion is stabilized by actin polymerization and mediates migration [6]. In addition it was previously demonstrated that *Aqp5*-KO mice show increased bacterial load after infection but decreased NFκB expression and mucus secretion compared to WT mice [10]. To this end, a decreased AQP5 expression may decrease immune cell migration and may subsequently alter the inflammatory chain during sepsis. The migration of neutrophils is essential in immune response during sepsis [11] and its regulation is critical for sepsis outcome as sufficient neutrophil migration is critical for pathogen clearance but excessive neutrophil migration is associated tissue damage and subsequent organ dysfunction [12]. In addition regulation of AQP5 expression in neutrophils has not been studied yet and was therefore addressed with this study.

Accordingly, we prospectively tested the following hypotheses:

1. The survival rate in *Aqp5*-KO mice is different than in wild type mice after lipopolysaccharide induced inflammation.
2. The AQP5 expression and the AQP5-1364A/C genotype is associated with impaired cell migration.

## Methods

### Mice

Experiments were conducted in accordance with German and local governmental regulations, complying with both the European Community and the American Physiological Society and the Guide for the Care and Use of Laboratory Animals (National Institute of Health publication 85-23, revised 1996) guidelines. *Aqp5* (−/−) [knockout (KO)] and wild type recombinant inbred from 129SvJ/Black Swiss mice were provided by Prof. Anil Menon, Cincinnati, and generated as previously described [13].

### Genotyping of mice

Mice were genotyped with mouse DNA extracted from an ear biopsy as described previously [14].

### Animal preparation

We investigated Aqp5-KO (−/−) and wild type (+/+) mice after an intraperitoneal (i.p.) injection of either* Escherichia coli* lipopolysaccharide (LPS, 20 mg/kg in saline [15], serotype O127:B8, Sigma-Aldrich, Taufkirchen, Germany) or an equivalent amount of pyrogen-free saline. After 4.5 h and 24 h post LPS/saline injection 100 µl retro orbital blood was collected. The serum layer was removed after clotting and centrifugation and stored at −80 °C. IL-10 and TNF-α concentrations were determined using Legend Max Elisa Plates (BioLegend, San Diego, CA, USA). The survival and the state of health (score sheet) of the mice were observed every 6 h. In the score sheet points (zero for no abnormality and 20 for worst case) were given for body weight, general condition, behavior, clinical findings and secretions. Due to these points the disease score was calculated (Additional file 1).

Eight *Aqp5*-KO (−/−) and eight wild type (+/+) mice were killed 3 h after LPS or saline injection by cervical dislocation to study neutrophil migration. Peritoneal lavage and lungs were harvested and right lungs were snap frozen whereas left lungs were stored in formalin and paraffin-embedded (Paraplast Xtra-plus, Sigma-Aldrich, Munich, Germany).

### MPO activity assay

Myeloperoxidase (MPO) activity was measured as a marker for neutrophil infiltration. The right lungs were homogenized in buffer containing 10 mM NEM (Sigma-Aldrich, Taufkirchen, Germany) and then solubilized in buffer containing 0,5% HTA-Br. MPO activity was determined by the use of Myeloperoxidase activity assay kit (abcam, Cambridge, UK). In addition protein amount was determinated using RotiQuant universal (Roth, Karlsruhe, Germany).

### Flow cytometric analysis of peritoneal lavage

For flow cytometry (FACS) cell suspensions were collected by a lavage of the peritoneum with 5 ml PBS containing 5 mM EDTA. For FACS analysis, cells were stained with the appropriate antibodies (APC antimouse Ly6g/Ly6C (Gr-1) clone RB6-8C5, BioLegend, San Diego, CA, USA; Antimouse CD11c FITC, clone N418, eBioscience, Frankfurt, Germany), after blocking FcRs with anti-CD16/32. Cells were analyzed on a BD FACSCanto II (Heidelberg, Germany). Data were analyzed with Flowing Software 2.

### Neutrophils staining in lung tissue with naphthol AS-d chloroacetate (NASDCL)-esterase

Naphthol AS-d chloroacetate (NASDCL)-esterase was used to detect neutrophils on tissue sections. Paraffin left lung sections (1–2 μm thick) were mounted on slides and de-paraffinized. Staining with naphtol AS-d chloroacetate staining solution (containing naphtol-AS-d-chloracetat in dimethyl sulfoxide dimethyl, pararosaniline and sodium nitrite) was performed for 30 min. Finally, the sections were counterstained with Mayer’s hematoxylin.

### Patients

The study was reviewed and approved by the Medical Faculty’s Ethics Committee of the Duisburg-Essen University. The samples used in this study have been used for several other studies before [3, 4, 9] thus no additional material had to be collected. Patients with sepsis were considered eligible when they fulfilled the 2001 American College of Chest Physicians/Society of Critical Care Medicine Consensus conference guidelines [16]. Patients with sepsis were enrolled on the day when the sepsis was first diagnosed and arterial and venous blood was sampled. Afterwards, blood tests, genotyping, cytokine determination, microbiology cultures, and RNA extraction were performed. Informed consent for all the patients was obtained from the patient’s guardian.

### Genotyping of patients and healthy volunteers

Genotyping was performed as previously described [7].

### RNA isolation and cytokine concentration measurements

RNA and cytokine extraction and measurement was performed as described [4].

### Immunochemistry

Neutrophils were isolated from 30 ml of venous blood obtained from four healthy donors after ethics committee approval (ethics committee of the Essen University Hospital, Essen, Germany) and a written informed consent. Immunostaining was performed with an AQP5 antibody [AQP5 G-19; sc-9890] as described previously [17].

### Stable transfection of jurkat T-cells

Stable transfection with Jurkat T-cells was performed using an AQP5_pReceiver EX-T1015-M09 vector (Human Full ORF-Clone, NM-001651, pReceiver-M09) or a pReceiverM09 (GeneCopeia, Rockville, MD, USA). The vector was linearized with ScaI (New England Biolabs, Ipswich, MA, USA) and transfected into Jurkat cells by electroporation using the Gene Pulser Xcell Electroporation System (BioRad, Munich, Germany). The cells were maintained in RPMI plus 10% FKS, plus Pen/Strep plus 1.1 mg/ml geniticin disulfate. Positive clones were selected using limiting dilution [18].

### Western blot

For protein extraction, stable transfected Jurkat cells or Chemotactic peptide (*N*-Formyl-l-methionyl-l-leucyl-l-phenylalanine, fMLP) stimulated HL-60 (10^−8^M fMLP for 6 h) promyelocyte cells were lysed with RIPA buffer and proteins were extracted by shaking at 4 °C. Western Blot was performed as described [19].

### Real-time PCR

RNA from blood cells of septic patients was extracted as described above and 0.5 µg RNA was used to synthesize cDNA. In addition RNA from fMLP stimulated HL-60 cells (10^−8^M for 6 h) was extracted and 1 µg RNA was used for cDNA synthesis. Primers for AQP5 and the housekeeping gene actin were used and the real-time PCR was performed as described [19, 20].

### Neutrophils migration assay

Blood samples (30 ml) from 8 AQP5-genotyped healthy volunteers (EDTA-Monovetten Sarstedt, Nümbrecht, Germany) were centrifuged at 500*g* for 30 min using 30 ml Polymorphprep (Fresenius Kabi, Oslo, Norway). Neutrophils were collected. Their purity was determined using “Scil Vet ABC” cell counter (Scil, Viernheim, Germany) and was above 90%. 5 * 10^5^ cells were deposited in 200 µl RPMI into the upper compartment of a filter migration assay system containing a polycarbonate membrane filter (5 µm pore size, BD, Heidelberg, Germany). The lower compartment contained 10^−8^ M fMLP (Sigma-Aldrich, Taufkirchen, Germany) in 500 µl RPMI or control media. The cells were incubated at 37 °C with 5% CO_2_ in air for 0.5 and 1.5 h in duplicate for each sample as described before [21]. The migrated cells were counted after 0.5 and 1.5 h using the MUSE Count & Viability Kit (Merck Millipore, Darmstadt, Germany). On the one hand total migrated cells (all cells which migrated to RPMI containing 10^−8^ M fMLP) and on the other hand target oriented migrated cells (difference between cells migrated to RPMI containing 10^−8^ M fMLP and spontaneously migrated cells to RPMI alone) were counted and calculated.

For the migration assay with Jurkat T-cells, stable transfected cells were used. Polycarbonate membrane filters were coated with 6.3 µg fibronectin and 5 * 10^5^ cells were deposited in 200 µl RPMI into the upper compartment. The lower compartment contained 100 ng/ml SDF-1α (PROSPEC, East Brunswick, NJ, USA) in 500 µl RPMI or control media. Cells were counted using the MUSE Count & Viability Kit after 4.5 h and again after 24 h.

### Statistical analysis

Continuous variables are summarized as boxplots. For the comparison of continuous variables of two independent groups, we used exact Wilcoxon–Mann–Whitney tests; for two dependent groups exact Wilcoxon signed-rank tests. The time-to-event survival variable was summarized as the Kaplan–Meier estimator. The reported p-values are two-sided and within each panel adjusted for multiplicity by Bonferroni’s correction. A p value of <0.05 was regarded as statistically significant. All statistical analyses were done using GraphPad Prism 6 (La Jolla, CA, USA) or IBM SPSS Statistics 21.

## Results

### Survival of *Aqp5*-KO and wild type mice after intraperitoneal LPS injection

**Fig. 1:**
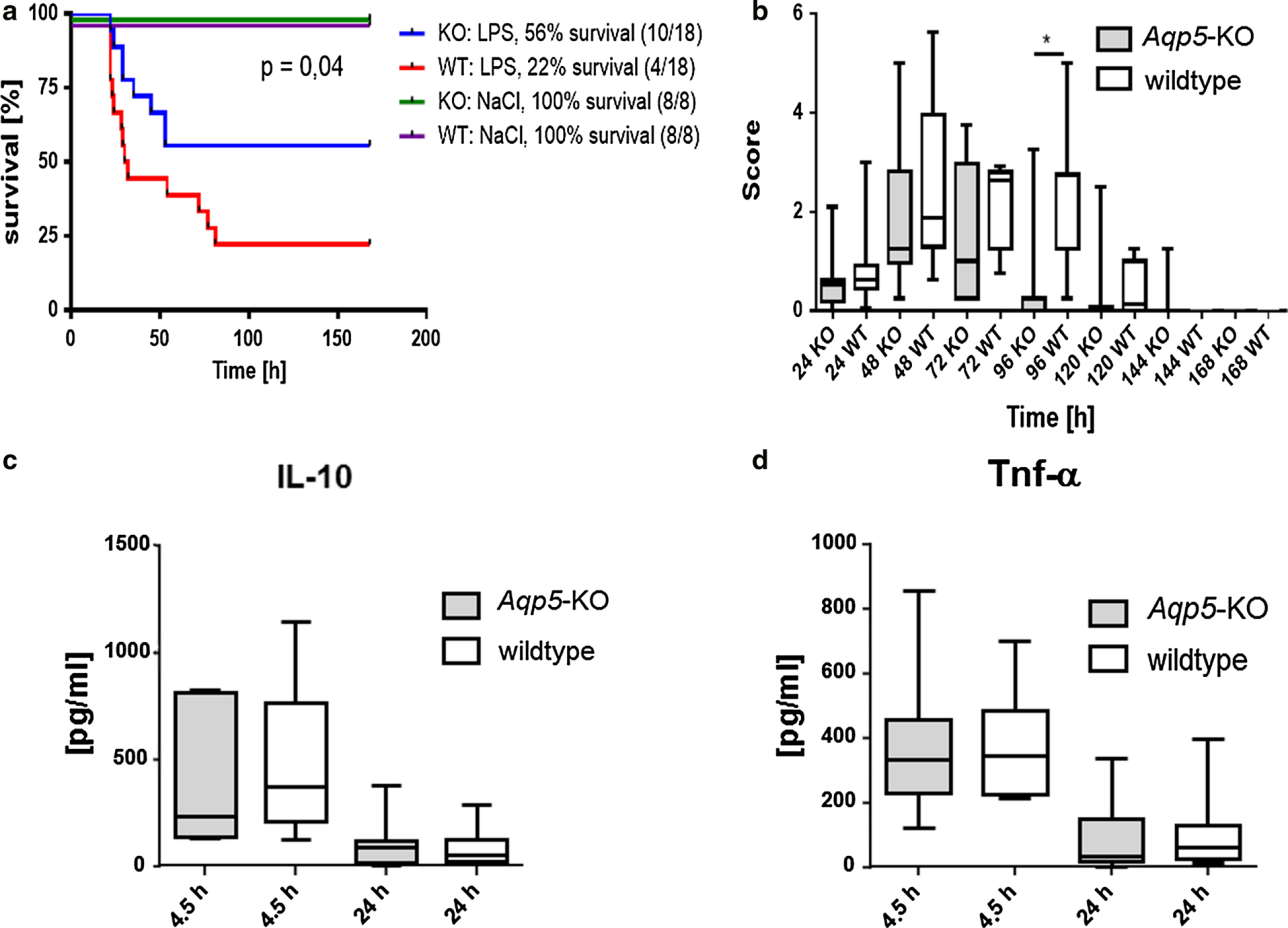
Survival, disease score and cytokine serum concentrations of *Aqp*5-knockout and wild type mice after intraperitoneal lipopolysaccharide injection. **a** Kaplan–Meier plot of overall survival of *Aqp5*-KO and WT mice after i.p. application of 20 mg/kg bodyweight lipopolysaccharide (LPS) or saline (NaCl) (n = 18 per group, saline: n = 8). 56% (10/18) of the *Aqp5*-KO mice but only 22% (4/18) of the WT mice survived for 7 days (p = 0.04). **b** Disease score recorded at different time points after LPS injection. WT mice showed a significantly increased disease score compared to Aqp5-KO mice (p = 0.028) after 96 h. **c**, **d** Interleukin-10 and tumor necrosis factor alpha serum concentrations of *Aqp5*-KO and WT mice measured 4.5 and 24 h after intraperitoneal LPS injection. IL-10 concentrations (**c**, n = 7, 9, 10, 9); TNF-alpha concentration (**d**, n = 13, 12, 12, 11) No differences were detected in concentrations of these cytokines between Aqp5-KO and WT mice. p ≫ 0.05 for all comparisons between *Aqp5*-KO and WT mice. **b**–**d** Data are shown as *boxplots* (min to max)

Seven-day survival following LPS injection differed between *Aqp5*-KO and WT mice. Fifty-six percent (10/18) of the *Aqp5*-KO mice but only 22% (4/18) of the WT mice survived for seven days (Fig. 1a). Overall, WT mice had a higher risk of death (fully adjusted hazard ratio (HR):2.48, 95% CI 1.04–5.90) compared to *Aqp5*-KO mice. The administration of LPS caused a quick onset of clinical signs of a systemic inflammatory response, such as reduced motor activity, lethargy, shivering, and weight loss. In general, these clinical signs were more severe in wild type than in *Aqp5*-KO mice. After 96 h, WT mice showed a significantly increased disease score compared to KO mice (Fig. 1b). To clarify if different cytokine expressions might be responsible for differences in survival in wild type and KO mice, we examined serum concentrations of tumor necrosis factor alpha (TNF-α), as one of the early pro-inflammatory cytokines [22], and of interleukin-10 (IL-10), as an important anti-inflammatory cytokine in sepsis [23]. We did not observe evidence for differences in cytokine concentrations of IL-10 and TNF-α in the blood of the *Aqp5*-KO and WT mice (Fig. 1c, d).

### Migration of neutrophils in mice tissues

**Fig. 2:**
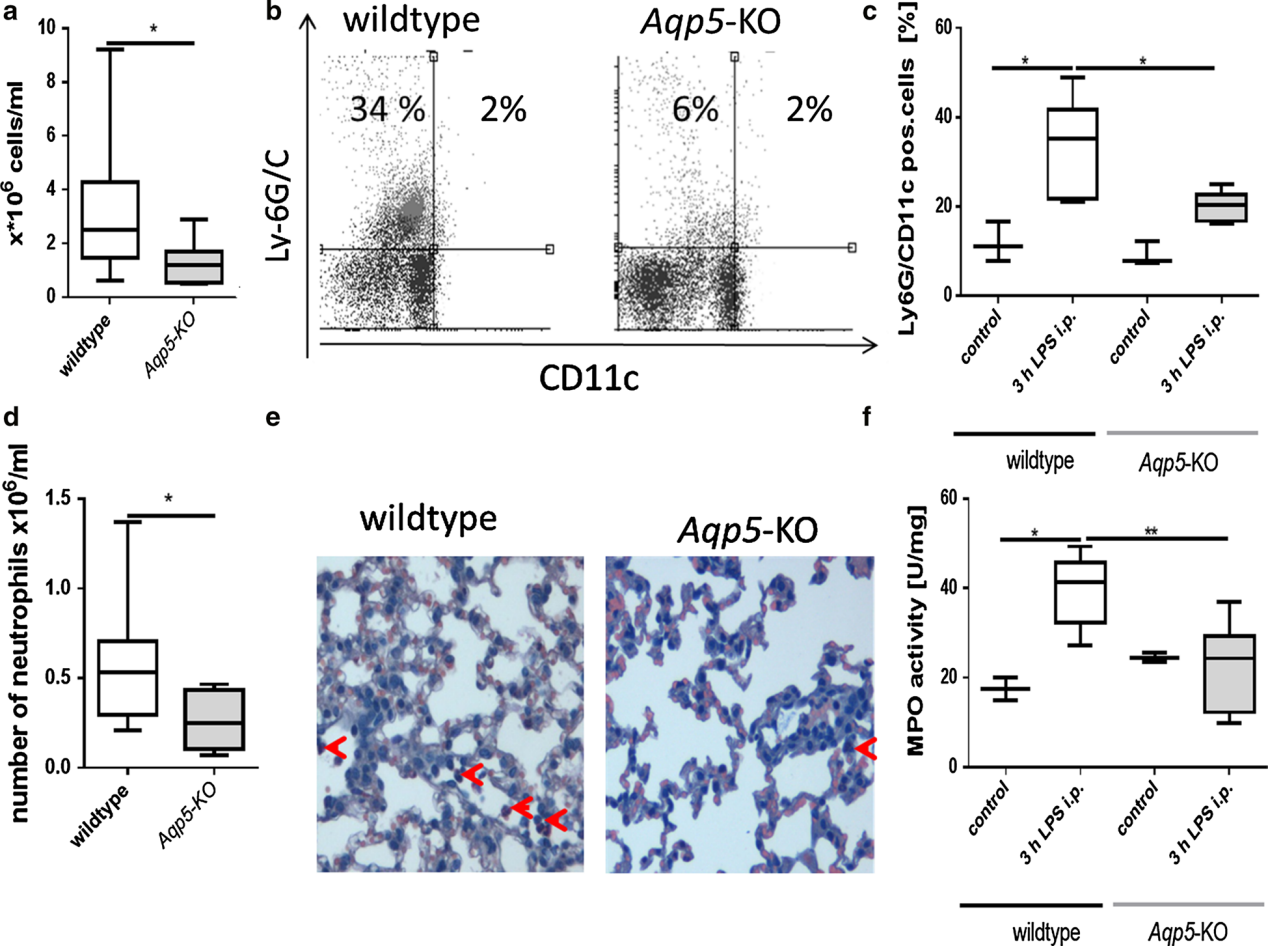
Neutrophil migration in peritoneum and lung 3 h after LPS or saline injection i.p. (20 mg/kg). **a** Total cell count in the peritoneum was 1.2 * 10^6^ cells/ml in median in *Aqp5*-*KO* mice compared to 2.5 * 10^6^ cells/ml in WT mice (n = 8; p = 0.04). **b** Representative* dot plots* show flow cytometric analysis of CD11c and Ly6G/C positive cells in the peritoneum. CD11c Ly6G/C positive cells were increased in WT mice compared to *Aqp5*-KO mice after LPS injection **c** Percentage of CD11c Ly6G/C positive cells in the peritoneum. In WT mice (n = 4) percentage of CD11c Ly6G/C positive cells significantly increased after LPS injection compared to saline injection (p = 0.017) and was higher compared to Aqp5-KO mice (n = 4) (p = 0.035). **d** Total cell count of CD11c Ly6G/C positive cells (n = 8; p = 0.04). **e** Representative NASDCL-Esterase staining of formalin fixed lung tissues (40× magnification). WT mice (n = 2) showed higher number of neutrophils compared to *Aqp5*-KO (n = 2) mice (*red arrows*) after 3 h LPS injection **f** Myeloperoxidase (MPO) activity in mice lungs. In WT mice (n = 4) MPO activity significantly increased after LPS injection compared to saline (p = 0.04) and was higher compared to Aqp5-KO mice [n = 4 (p = 0.004) **f**]. Data are shown as *boxplots* (min to max; n = 8 in total)

First we analyzed total cell count in the peritoneal lavage of *Aqp5*-KO and WT mice. *Aqp5*-KO mice had significant deceased cell count in the lavage compared to WT mice (Fig. 2a). Furthermore we analyzed the percentage of CD11c Ly6G/C positive cells in the peritoneum of *Aqp5*-Ko and WT mice at 3 h after i. p. LPS administration compared to saline injected control mice with flow cytometry (Fig. 2b). In WT mice percentage of CD11c Ly6G/C positive cells significantly increased after 3 h LPS compared to saline (Fig. 2c), whereas no significant increase was detected in *Aqp5*-KO mice 3 h after LPS compared to saline injection. Hence, we found a significantly higher amount of CD11c Ly6G/C positive cells in the peritoneum, of WT mice compared to *Aqp5*-KO mice (Fig. 2d) after LPS administration.

In the lungs of WT mice a higher number of neutrophils were detected compared to *Aqp5*-KO mice after NASDCL-Esterase staining on fixed lung samples (Fig. 2e). In line with this MPO activity increased in lungs of WT mice after 3 h LPS in comparison with saline injection, but not in *Aqp5*-KO mice (Fig. 2f). Therefore 3 h after LPS administration WT mice showed significantly higher MPO activity compared to *Aqp5*-KO mice (Fig. 2f) in lungs.

### AQP5-dependent cell migration of a human cell line

**Fig. 3:**
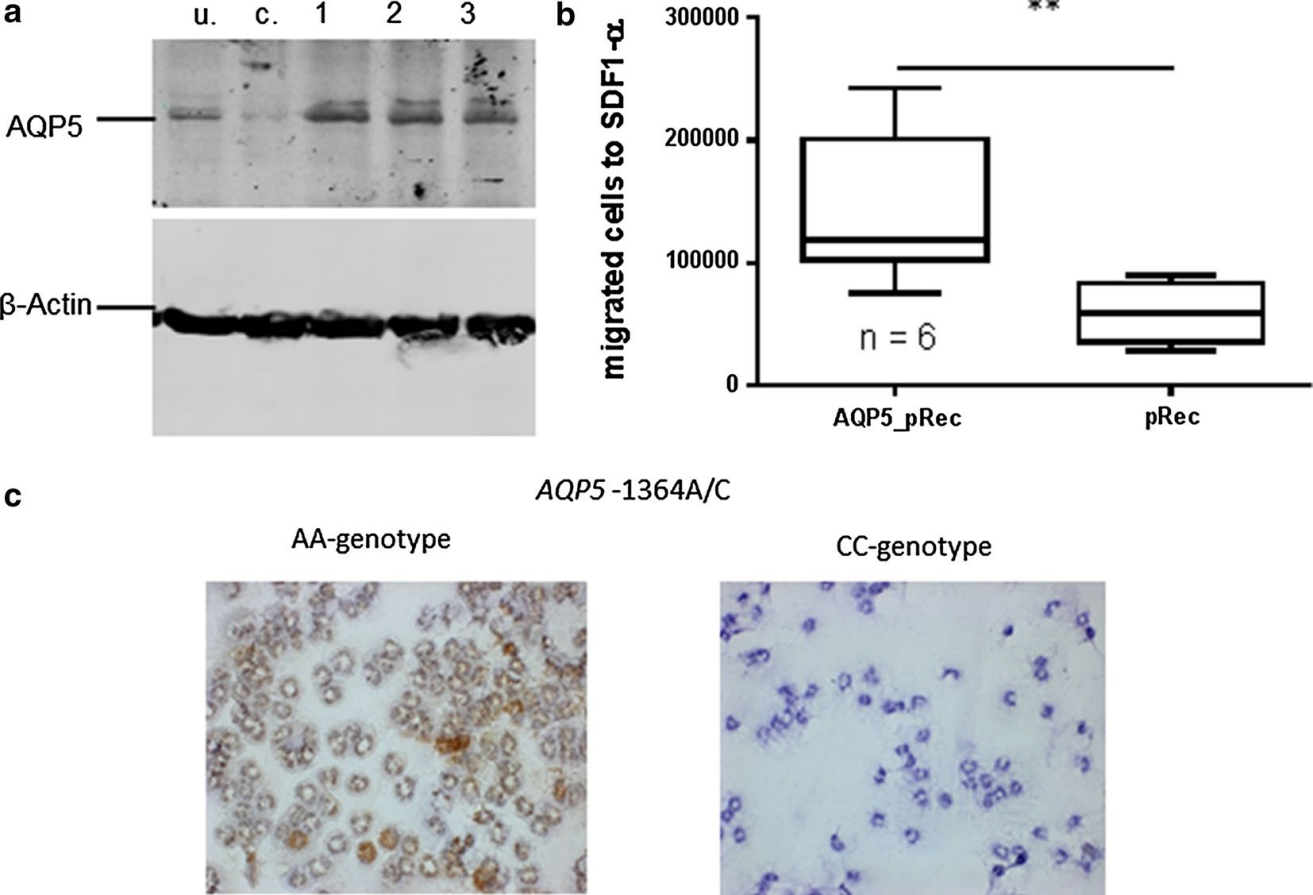
Western blot and migration assay of transfected Jurkat-cells and AQP5-immunostaining of neutrophils obtained from healthy donors **a** Western blot from whole cell lysates of Jurkat-cells (*lane u* untransfected cells, *lane c* transfected with control plasmid, *lanes 1*–*3* AQP5_pReceiver transfected Jurkat cells) with AQP5 and ACTB antibody; As indicated by the stronger band in *lanes 1*–*3*, Jurkat cells could successfully be stable transfected; **b** Migration assay performed with cell culture inserts utilizing transfected Jurkat cells stimulated with SDF-1α and counted after 22 h (n = 6, 6); AQP5 overexpressing (AQP5_pRec) cells showed increased migration (p = 0.009) compared to control cells (pRec). **c** Representative examples of immunostaining with AQP5 antibody of neutrophils obtained from two healthy AA genotype carriers showing a considerable amount of AQP5 protein (*brown precipitate*). In neutrophils of two CC genotype carriers no clear AQP5 protein expression is detected. Every staining was performed in duplicate and eight random photographs were taken from each slide, 40× magnification

We used the Jurkat cell line for several reasons: (1) Jurkat cells can be transfected by routine protocols [28], (2) migration assay for Jurkat cells is well established using the chemoattractant SDF-1α [29], and (3) we could address AQP5 s impact on granulocytes’ (neutrophils) and lymphocytes’ (Jurkat) migration. As shown in Fig. 3a and validated by Western blot, Jurkat cells could be successfully stable transfected with an AQP5 overexpression vector and with a control vector. AQP5 transfected Jurkat cells showed a 2.4-fold increase in total cell migration compared to cells transfected with the control vector (Fig. 3b).

### Genotype-dependent AQP5 expression and migration of human neutrophils

**Fig. 4:**
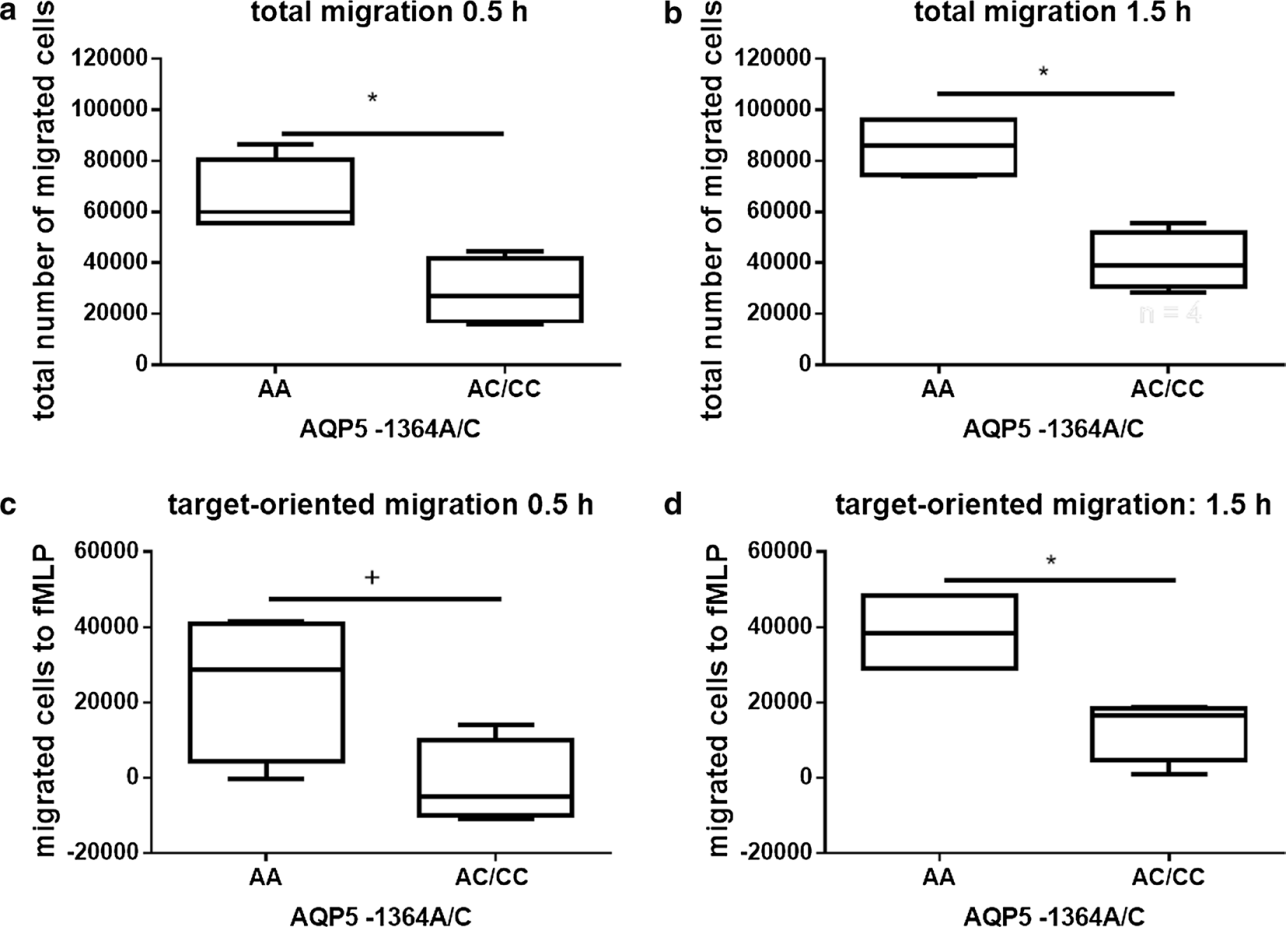
Migration assay of human primary neutrophils. Migration assays of human primary neutrophils unstimulated or fMLP (target) stimulated using cell culture inserts. Migrated cells were counted after 0.5 and 1.5 h (n = 4). Neutrophils from AA-genotype carriers showed target-oriented migration already after 0.5 h (**a** p = 0.029; **c** p = 0.056). Neutrophils from AC/CC-genotypes did not demonstrate target-oriented migration until 1.5 h, and, when compared to AA-genotype carriers, the number of migrated cells was threefold less than the AC/CC-genotype carriers (**b** p = 0.029; **d** p = 0.029)

**Fig. 5:**
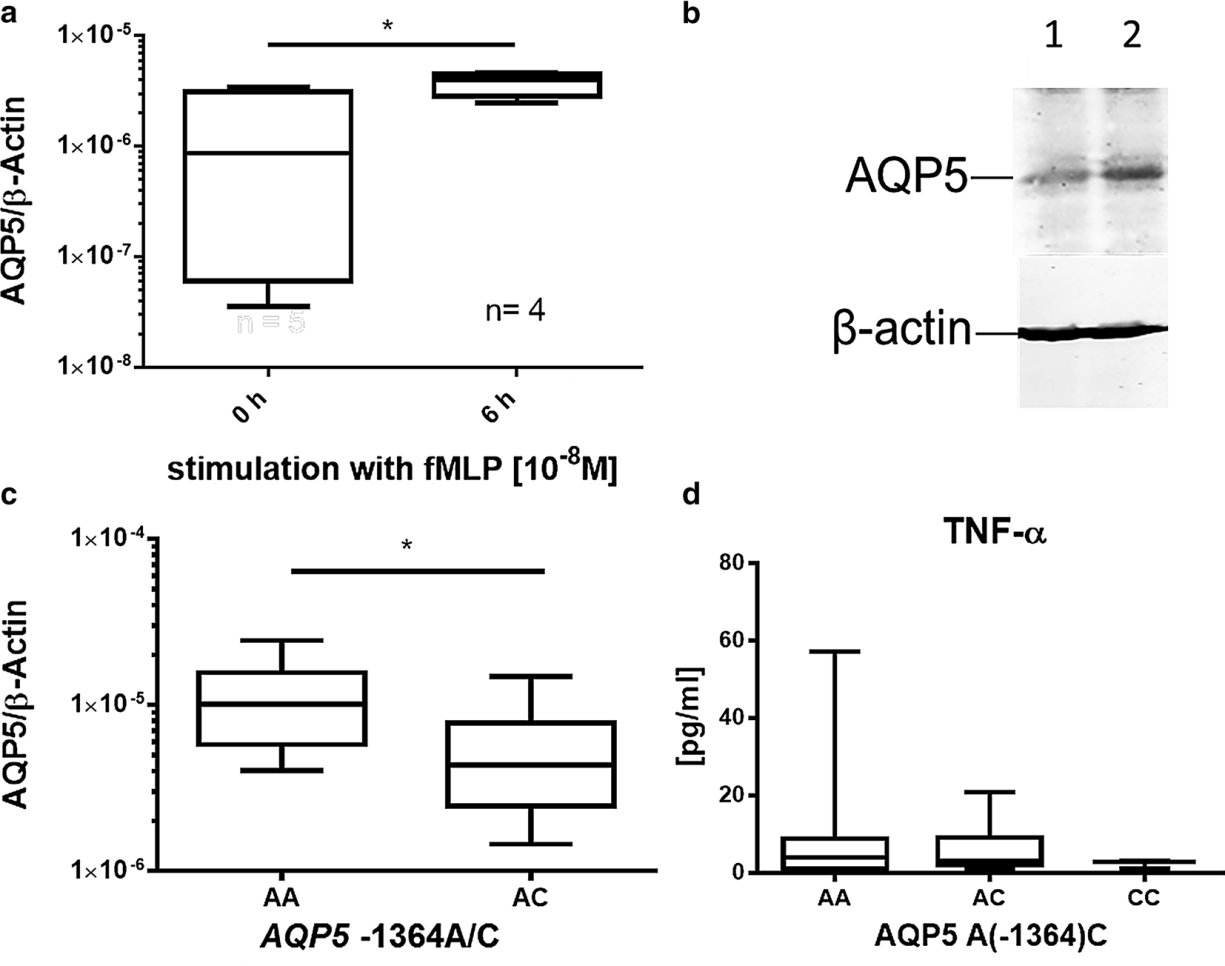
AQP5 expression in HL-60 promyelocytes after fMLP administration and in whole blood of septic patients. **a** AQP5 mRNA expression in HL-60 cells stimulated with 10^−8^ M fMLP for 6 h. FMLP significantly increased AQP5 expression (n = 5; p = 0.03). **b** AQP5 protein expression in fMLP stimulated HL-60 cells for 6 h. AQP5 protein amount is increased in fMLP (10^−8^ M)-stimulated cells (*lane 2*) compared to control (*lane 1*). **c** AQP5 RNA expression measured with RT-PCR in whole blood of septic patients (n = 14, 11); AA-genotype carriers showed an increased AQP5 mRNA expression compared to AC-genotype carriers (p = 0.025). No RNA was available from CC genotype carriers. **d** TNF-alpha serum concentrations of septic patients. No differences could be detected between genotypes (p ≫ 0.05); Data derive from serum of septic patients [(n = 65, 33, 3) **a**–**d**], data are shown as *boxplots* (min to max)

Neutrophils from healthy donors with AA genotype showed a considerable amount of AQP5 protein while no clear AQP5 protein expression could be detected in CC genotype carriers (Fig. 3c). Neutrophils of AA-genotype carriers already showed target-oriented migration after 0.5 h, while neutrophils from AC/CC-genotypes did not (Fig. 4a, b). After 1.5 h, neutrophils from AC/CC-genotype carriers also showed target-oriented migration, but the amount of migrating cells was threefold lower than in AA-genotype carriers (Fig. 4c, d). In addition, incubation of the neutrophil cell line HL-60 with the chemotactic peptide fMLP increased the *AQP5* mRNA expression (Fig. 5a). In line with that an increase in AQP5 protein expression was also evident by Western blot (Fig. 5b).

### AQP5 expression and cytokine concentrations in septic patients

To conclude, we examined if *AQP5* expression in septic patients differs between A and C-allele carriers as we showed it in neutrophils of healthy donors. The *AQP5* mRNA expression was greater in the blood of septic patients carrying the *AQP5* AA genotype compared to those carrying the AC genotype (Fig. 5c). In addition, TNF-α concentration in the blood of septic patients was determined depending on AQP5 genotype. Again as in mice (Fig. 1d), there was no evidence for *AQP5*-genotype dependent differences in TNF-α concentration (Fig. 5d).

## Discussion

This study suggests that AQP5 might be an important protein in LPS induced systemic inflammation and identifies the influence of AQP5 expression on immune cell migration as a potential mechanism by which the AQP5 expression affects survival in sepsis. Furthermore, we showed that the C-allele, associated with decreased AQP5 expression and increased survival from severe sepsis, is associated with a decreased neutrophil cell migration in vitro. Thus, the link between the *AQP5*-1364A/C polymorphism and sepsis survival could be due to AQP5 expression and its impact on neutrophil cell migration. Less cell migration can cause less tissue damage caused by infiltrating neutrophils and pro-inflammatory mediators [12, 24]. Another possible mechanism which was not examined in this study is the potential impact of AQP5 expression on neutrophil maturation. If AQP5 influences maturation the impact on cell migration would only be indirectly but it would still have the same impact on course of sepsis.

Despite great efforts in research and clinical treatment made in the last 30 years, the sepsis mortality remains unabatedly high [25]. Accordingly, new approaches are urgently needed. Our previous association study suggested that AQP5 seems to be a key protein in severe sepsis. Substitution of C for A at position −1364 was associated with decreased AQP5 protein expression and was a strong and independent prognostic factor for decreased 30-day mortality in patients with severe sepsis [9]. Therefore this study is in line with our previous study, which also showed a twofold decreased AQP5 mRNA and protein expression in C-allele carriers compared to A-allele carriers [7]. Our investigation of *Aqp5*-KO mice in comparison with WT mice now confirms our hypothesis that the AQP5 expression itself has a particularly strong influence on the mortality of sepsis and that this influence is associated with decreased neutrophil migration.

Findings from Vassiliou et al. support our results [19, 26] that aquaporins seem to be important target proteins in sepsis, as they identified AQP1 as a gene that is up-regulated in LPS induced inflammation. Importantly, an AQP1 upregulation not only took place in lung tissue [19] but also in leukocytes. Vassiliou et al. showed that the AQP1 expression induced by LPS was associated with increased cell membrane permeability [26]. In our study, we examined cell migration and showed that increased AQP5 expression is accompanied by increased neutrophil migration. The precise mechanism of AQP5 induced immune cell migration has not been clarified yet. Recently the mechanism of AQP9 induced neutrophil migration could be elucidated [27] and a similar mechanism was described for AQP1 [6]. Basing on these findings we can speculate on the mechanism of AQP5 induced cell migration. AQP5 could be phosphorylated by fMLP pathway and then translocated to the plasma membrane. Furthermore AQP5 expression could be increased by fMLP pathway, possibly via the transcription factor nuclear factor of activated T cells 1 (NFAT1) [28]. Increased AQP5 expression and localization of AQP5 to the plasma membrane leads to an influx of water and creation of a lower concentration of actin monomers which directs a flow of actin monomers to the site. Actin monomers polymerize and form a stable lamellipodium which leads to cell migration [6].

In this study, we also showed that AQP5 overexpression in the Jurkat lymphocyte cell line enhances SDF-1α induced migration. Interestingly, although SDF-1α is found ubiquitously throughout the body, lymphocytes from septic patients show increased migration towards SDF-1α [30].

We also demonstrate that fMLP induces AQP5-dependent neutrophil cell migration through a membrane. The process seems to amplify itself as we showed that fMLP increases AQP5 expression in the promyelocyte cell line HL-60. Neutrophil cell migration is an important mechanism occurring in sepsis [31, 32]. On the one hand, a decrease in neutrophil cell migration to the infected organs is associated with a bad outcome of sepsis, due to incomplete bacteria eradication [33]. On the other hand, neutrophils release large amounts of proteases and reactive oxygen species, which not only kill bacteria but also damage host tissues [34]. Therefore, an overwhelming neutrophil cell migration to the tissues can cause multiple organ failure, which leads to death [34].

Limitations of our study should be mentioned. To our surprise, we could not demonstrate in our mouse model of LPS induced inflammation that *Aqp5*-KO is associated with a decreased cytokine concentration. The lack of a genotype dependent effect in cytokine concentrations could be due to the wide variability in cytokine production, which is acknowledged both in mice [35] and humans [36, 37], and cytokine secretion in itself as well as the response to corticoids is influenced by genetic variations [3, 4]. In contrast to humans mice are inbreed littermates with no genetic variation and cytokine differences should have been detected. Other studies could detect differences in TNF-α expression between KO and wild type mice of other genes [38]. Therefore AQP5 might not influence TNF-α pathway and other inflammatory proteins might be responsible for the differences occurring in *Aqp5*-KO and WT animals.

## Conclusion

In summary, the *AQP5* genotype and AQP5 protein expression seem to alter neutrophil cell migration and may influence survival in sepsis by altering neutrophil cell migration. Therefore AQP5 might be a key protein in inflammation and depict a novel target for developing sepsis therapeutics.

## Additional files

**Additional file 1.** Score sheet for monitoring animal health.

**Additional file 2.** Raw data from the figures are available as an excel file.

## Abbreviations

AQP: aquaporin
KO: knockout
WT: wild type
LPS: lipopolysaccharide
TNF: tumor necrosis factor
fMLP: chemotactic peptide
IL: interleukin
