# Supplementary material for: AQP5-1364A/C polymorphism and the AQP5 expression influence sepsis survival and immune cell migration: a prospective laboratory and patient study
Source: J Transl Med. 2016 Nov 21;14:321. doi: 10.1186/s12967-016-1079-2 (PMC5117689; doi:10.1186/s12967-016-1079-2)
Supplement: Supplementary file 1 — Additional file 1. Score sheet for monitoring animal health. [file 12967_2016_1079_MOESM1_ESM.doc]

Score sheet zum Antrag: Untersuchung des Überlebens bei schwerer Sepsis bei AQP5 -/- Mäusen im Vergleich zu APQ +/+“; Antragsteller Dr. Adamzik

| **Beobachtung** | **Punktebewertung** |
| --- | --- |
| I Körpergewicht   - 0-5% Gewichtsveränderung - Gewichtsreduktion 5-20% - Gewichtsreduktion > 20% | 0  5  20 |
| II Allgemeinzustand   - Fell glatt, glänzend; Körperöffnungen sauber, Augen klar, glänzend - Struppiges/schmutziges Fell, verklebte oder feuchte Körperöffnungen, unnormale Haltung, Augen trüb, hoher Muskeltonus, geringgradige Dehydration (Hautfalte verstreicht verzögert), schnelle oder abdominale Atmung - Verkrampfungen, Lähmungen (Rumpfmuskulatur, Extremitäten), Atemgeräusche (schwerfällige Atmung), hochgradige Dehydration (Hautfalte bleibt stehen), dauerhaft gekrümmte Körperhaltung, schmutziges Fell, stark verklebte Augen/Nase, Kotverschmutzung | 0  10  20 |
| III Spontanverhalten   - normales Verhalten (Schlafen, Reaktion auf Käfigbewegungen und Berührung, Neugier, Sozialkontakte) - geringe Abweichung vom Normverhalten - ungewöhnliches Verhalten, eingeschränkte Motorik oder leichte Hyperkinetik - Selbstisolation, Lethargie; ausgeprägte Hyperkinetik bzw. Verhaltensstereotypien; Koordinationsstörungen, Fehlende Futter- oder Nahrungsaufnahme - Schmerzlaute beim Ergreifen; Selbstamputation (Automutilation), Unfähigkeit der Nahrungsaufnahme | 0  1  5  10  20 |
| IV Klinischer Befund   - Atmung normal, Extremitäten warm - Atemfrequenz merklich erhöht, Erhöhung der Herzfrequenz - Schnappatmung; - Bewusstseinsverlust | 0  10  20  20 |
| V Ausscheidungen   - normal unverändert - leichte Veränderung in Form und Konsistenz - mäßiger Durchfall oder trockener Kot, abnormaler Urin (Menge/Farbe) - unkontrollierter Durchfall, blutiger Kot, kein Urin/Kot oder inkontinent | 0  5  10  20 |
| Bewertung; Maßnahmen  0-9 Punkte:  Keine oder geringe Belastung; Tier wird weiterhin täglich kontrolliert  10-19 Punkte:  Mittelgradige Belastung, engmaschigere Überwachung (alle 6 Stunden); Tier wird schmerzfrei getötet, wenn nach 2 Tagen keine Besserung eingetreten ist  20 Punkte oder mehr:  Hochgradige Belastung, Versuch abbrechen, Tier wird schmerzfrei getötet | |
